# Compensate for or Minimize Matrix Effects? Strategies for Overcoming Matrix Effects in Liquid Chromatography-Mass Spectrometry Technique: A Tutorial Review

**DOI:** 10.3390/molecules25133047

**Published:** 2020-07-03

**Authors:** Manuela Cortese, Maria Rosa Gigliobianco, Federico Magnoni, Roberta Censi, Piera Di Martino

**Affiliations:** 1 Laboratory of Mass Spectrometry, University of Camerino, School of Pharmacy, Via S. Agostino, 62032 Camerino, Italy; manuela.cortese@unicam.it; 2 Laboratory of Pharmaceutical and Biotechnological Sciences, University of Camerino, School of Pharmacy, Via S. Agostino, 62032 Camerino, Italy; maria.gigliobianco@unicam.it (M.R.G.); roberta.censi@unicam.it (R.C.); 3 Institute for Material Research & Innovation, University of Bolton, Deane Road, Bolton BL3 5AB, UK; F.Magnoni@bolton.ac.uk

**Keywords:** matrix effect, HPLC-MS, method validation

## Abstract

In recent decades, mass spectrometry techniques, particularly when combined with separation methods such as high-performance liquid chromatography, have become increasingly important in pharmaceutical, bio-analytical, environmental, and food science applications because they afford high selectivity and sensitivity. However, mass spectrometry has limitations due to the matrix effects (ME), which can be particularly marked in complex mixes, when the analyte co-elutes together with other molecules, altering analysis results quantitatively. This may be detrimental during method validation, negatively affecting reproducibility, linearity, selectivity, accuracy, and sensitivity. Starting from literature and own experience, this review intends to provide a simple guideline for selecting the best operative conditions to overcome matrix effects in LC-MS techniques, to obtain the best result in the shortest time. The proposed methodology can be of benefit in different sectors, such as pharmaceutical, bio-analytical, environmental, and food sciences. Depending on the required sensitivity, analysts may minimize or compensate for ME. When sensitivity is crucial, analysis must try to minimize ME by adjusting MS parameters, chromatographic conditions, or optimizing clean-up. On the contrary, to compensate for ME analysts should have recourse to calibration approaches depending on the availability of blank matrix. When blank matrices are available, calibration can occur through isotope labeled internal standards and matrix matched calibration standards; conversely, when blank matrices are not available, calibration can be performed through isotope labeled internal standards, background subtraction, or surrogate matrices. In any case, an adjusting of MS parameters, chromatographic conditions, or a clean-up are necessary.

## 1. Introduction

Liquid chromatography-mass spectrometry techniques figure among the most powerful and useful analytical instruments for quantification of organic components in complex mixtures in environmental studies [1], food quality and composition research [2], and bioanalytical and pharmaceutical fields [3,4]. Because of their sensitivity and specificity, especially in tandem mass mode, they are the techniques of choice in most private and government quality control laboratories [5,6], that apply validated analytical methods.

Despite the advanced features of HPLC-MS technology, the validation process is not easily performed, especially because of its susceptibility to matrix effects (MEs) [7]. In analytical chemistry, ME is defined as “*the combined effects of all components of the sample other than the analyte on the measurement of the quantity. If a specific component can be identified as causing an effect then this is referred to as interference*” [8]. When a mass spectrometer is used for quantitation, especially with atmospheric pressure ionization (API) interfaces, the interference species can alter the ionization efficiency in the source, when they co-elute with the target analyte. These effects may cause ionization suppression or ionization enhancement [9]. The most common API sources are electrospray ionization (ESI), and atmospheric pressure chemical ionization (APCI). The mechanisms related to ion suppression in both of them are extensively reported by Trufelli et al. [10], who distinguish between the mechanisms occurring when the charged analyte is formed in the liquid phase or in the gas phase [11,12,13]. In particular, in ESI, the ionization occurs in the liquid phase and then the charged analyte is transferred to the gas phase. In APCI, the analyte is transferred in the gas phase as a neutral molecule and ionized in the gas phase by chemical ionization. Most of the mechanisms causing ion suppression in ESI in the liquid phase are not present in APCI. This is the main reason that APCI is sometimes less prone to MEs. Instead, ionization enhancement might be related to the analyte’s relative affinity with the droplet surface, or to the overlap of interferences responding at the same MS signal chosen for the target analyte.

Interference characteristics in complex matrices may range from hydrophilic species, like inorganic salts in urine, to hydrophobic molecules, like proteins, amino acids and phospholipids in plasma and oral fluids. The presence of these compounds can strongly influence method ruggedness, affecting precision and other parameters such as accuracy, linearity, and limits of quantification and detection, which are all crucial factors that are evaluated during a validation process [14].

The extent of ME is widely variable and unpredictable. It can be strictly dependent on the interactions between the analyte and the interfering co-elution [15], or it may be not specific, or a result of cross-contamination by previous samples and high concentrated standards: the same analyte can give different MS responses in different matrices, and the same matrix can affect different target analytes in a different way.

Simple and common practice for minimizing MEs is the use of a divert valve that is able to switch the flow coming from the column to the ionization source or waste, resulting in a less ion source contamination [16,17,18]. But, to achieve a complete elimination of MEs, a selective extraction should be planned and performed [19,20]. Recently, the development of molecular imprinted technology (MIP) should provide the analyst with new opportunities in terms of selective extraction, high recovery percentage and low MEs [21,22], but unfortunately this technology is not yet commercially available. Generally, the more similar the polarity between the target analytes and the matrix composition, the less chance there is for efficient and selective extraction step using the common extraction procedures. In this case, several ways to compensate for or overcome MEs are described in the literature. These alternatives will be the subject of subsequent chapters in this Review.

Based on the literature and our own laboratory experience, when sensitivity is not a crucial parameter, the choice of quantification strategy depends upon the availability of a suitable blank matrix, because it makes it possible to compensate for MEs in an easier way using a more standardized procedure. For quantitation of endogenous compounds, the surrogate matrix may have similar performance, even if it is necessary to demonstrate similar MS response of the analyte in both original and surrogate matrix [23]. The lack of a blank matrix corresponds to longer times of optimization, and the need to evaluate different techniques to reduce MEs. On the other hand, when sensitivity is a crucial parameter, the need for a pre-concentration step may lead to a cleaner sample, reducing MEs, but this step can also concentrate the co-eluting substances in the sample, resulting in comparable or worse MEs values.

**Figure 1:**
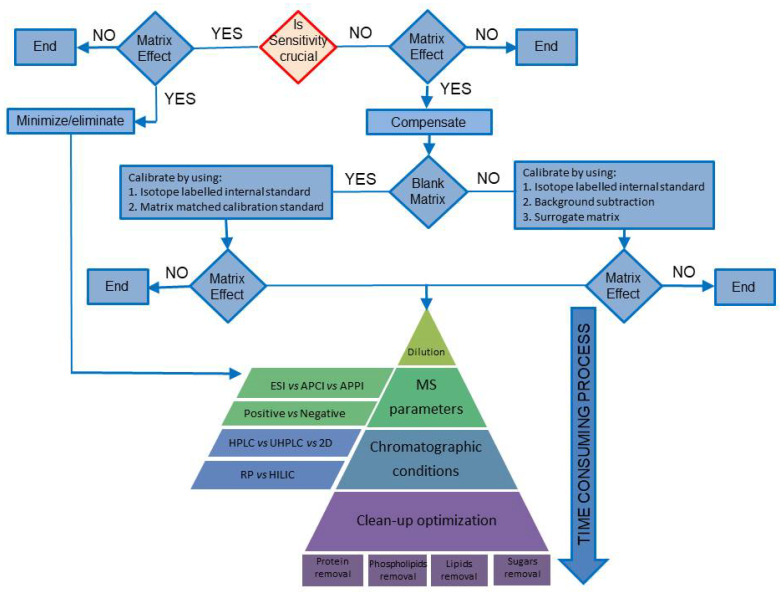
Flow chart of the reviewed methodologies to evaluate and overcome the matrix effect.

In this review, authors intend to offer a guideline to minimize/compensate ME, by describing various strategies in the development of a new analytical method, and by indicating the most suitable methods, in terms of accuracy and time required (Figure 1) [24].

## 2. Evaluation of MEs

The optimization of the MS parameters for each analyte by using the correspondent pure standard is the first step during the development of a new analytical method. Then, the evaluation of MEs should be the next step, and not just a step of the validation process in order to evaluate method performance. Early assessment of MEs could improve the final method in term of ruggedness, precision, and accuracy.

MEs may be assessed using three main techniques (Table 1): the post-column infusion system proposed by Bonfiglio et al. [25], the post-extraction spike method proposed by Matuszewski et al. [26], and its modification proposed by Romero-Gonzáles et al. [27] and Sulyok et al. [28], called “*slope ratio analysis*”.

**Figure 2:**
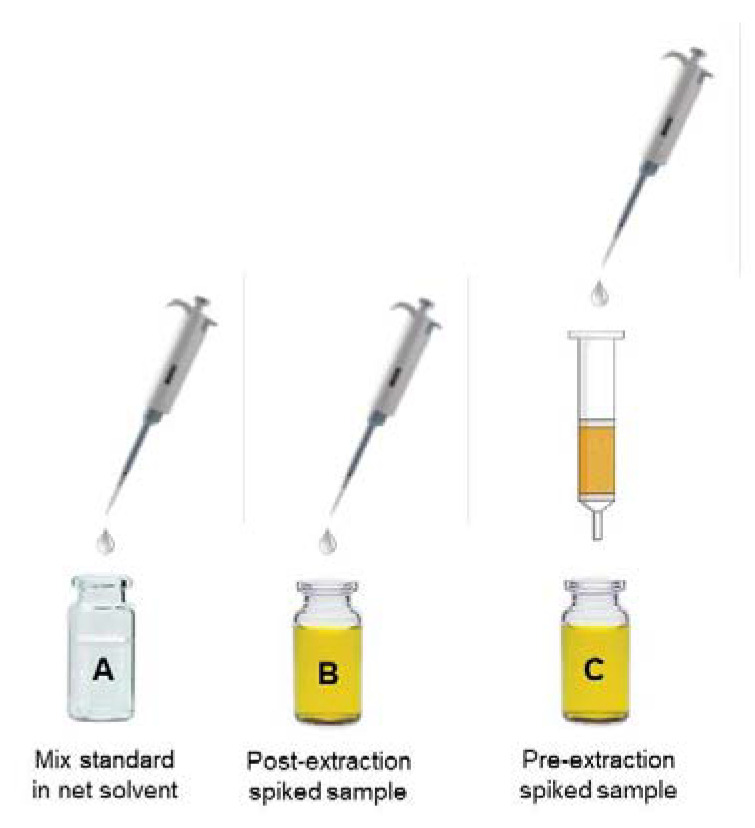
Qualitative evaluation of MEs by post-column infusion method on the compound (**A**). Comparison between two common protein precipitation agents on a blank plasma sample: (**B**) acetonitrile, and (**C**) perchloric acid. Acetonitrile generally gave less signal suppression among the entire chromatographic profile, and in particular in the elution zone of the compound A (unpublished data).

These three approaches give different, complementary, and important information about sample preparation and its effects on ME. Post-column infusion is the most suitable for an early qualitative approach to MEs evaluation, focusing on performance of the preparative step (Figure 2), while the post-extraction spike method, and the slope ratio analysis method are more related to the validation step and result in a quantitative evaluation of MEs for a single level or a range of concentrations. The following sections evaluate these three different approaches in depth.

### 2.1. Post-Column Infusion

**Figure 3:**
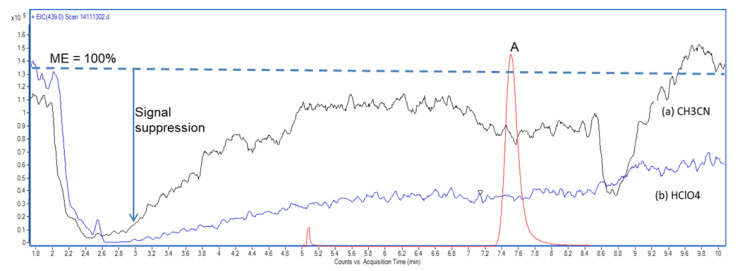
MEs evaluation by post-column infusion.

The post-column infusion method provides a qualitative assessment of MEs. A chromatographic plot identifies zones of retention times most likely to experience phenomena of ion enhancement or suppression [10]. The analysis is performed by injecting a blank sample extract through the LC-MS system, and a post-column infusion of the analyte standard through a T-piece [25,29] (Figure 3).

If a blank matrix is not available, the post column infusion can be performed using a labeled internal standard instead of the analyte standard [30]. ME can be assessed as suppression or enhancement of the analyte signal in specific regions of the chromatogram [7].

A good illustration of the practical importance of the post-column infusion method was offered by Stahnke and co-workers [31], who systematically compared the ME in LC-ESI/MS for 129 pesticides in 20 different plant matrixes, applying this method to assess the MEs throughout the duration of the entire chromatographic run, which allowed evaluation independently of a specific retention time. In addition, this approach is useful for understanding the mechanism involved in the ionization process, its relation with the molecule functional group and the matrix itself.

Rossman and co-workers conducted a MEs evaluation of 33 pharmaceuticals in biological matrices (urine and plasma) together with an environmental matrix (urban wastewaters) [32]. They concluded that most of the substances analyzed had a similar ME profile in the same matrix independently of their structure, while they had a different ME profile moving from urine to plasma, to an environmental matrix, a not uncommon situation. In contrast, six of the 33 molecules analyzed revealed signal enhancement independent of the analyzed matrix, a behavior found to be associated with the presence of at least one hydroxyl-group. Given these observations, the authors suggested that it is advisable to conduct constant and simultaneous correction of MEs during measurements.

The only way to achieve this goal is to use internal standards. In recent literature, the most common use of the post-column infusion is to evaluate how the preparative step reduce MEs [33,34]. Several other authors adopted this approach for MEs assessment [35,36,37]. In addition, it may be very useful to evaluate the influence of chromatographic conditions, and the role of mobile phase additives on the analyte response and on the MEs [10].

Unfortunately, the major limitation of post-column infusion is the laboriousness of the procedure, especially when multiresidue analysis has to be performed. In fact, any possible MEs have to be evaluated separately for each analyte and each chromatographic and sample extraction condition. In addition, it does not provide a quantitative evaluation of MEs, and it does not give information about the interference causing ion suppression, or the presence of endogenous compounds that share the transition and result in ion enhancement. The analyte is generally infused at a concentration greater than the limit of quantification (LOQ), and thus it is inefficient for highly diluted samples [38].

In addition, the concentration of the analyte should be in the analytical range being investigated, because otherwise a greater amount of analyte may interfere at the ion source, leading to incorrect results [39]. All these drawbacks make the post-column infusion method useful for particular investigation on MEs [32] or for developing quantitative analytical method on a few number of molecules, mainly for the optimization of the preparation step. It is not feasible for routine analytical laboratories, as it is a qualitative approach to MEs evaluation, and a quantitative estimation is necessary for the validation process.

### 2.2. The Matuszewski Post-Extraction Spike Method

In the post-extraction spike method, the response of the analyte in a standard solution is compared to that of the analyte spiked into a post-extraction sample at the same concentration. Deviations from the responses of the two solutions are identified as ion enhancement or suppression. The advantage of this approach over the previous one is that it provides a quantitative and more accurate assessment of MEs [40,41,42,50].

According to the precursor studies of Buhrman et al. [51], ion suppression can be estimated by Equation (1):

(1) $$\mathrm{ME}(\%)=100-\frac{B}{A}\times 100$$

where *A* represents the average peak area of a standard solution and *B* represents the average peak area of a plasma extract spiked at the same concentration of the standard. Using this formula, the ion enhancement corresponds to negative values of the ME (%). Matuszewki and co-workers [26] proposed another approach, introducing also the concept of process efficiency (PE), defined as the combination of MEs and analyte recovery from the matrix according to the extraction process. Many authors have adopted this evaluation method. For example, Wu et al. [43] assessed the MEs in the determination of trace levels of pharmaceuticals in seawater. Caban et al. [44] evaluated the MEs during the analysis of pharmaceuticals in environmental samples. Matuszewki et al. [26] evaluated the ME%, the recovery % (RE%), and the process efficiency % (PE%) by Equations (2)–(4), respectively:

(2) $$\mathrm{ME}(\%)=\frac{B}{A}\times 100$$

(3) $$\mathrm{RE}(\%)=\frac{C}{B}\times 100$$

(4) $$\mathrm{PE}(\%)=\frac{C}{A}\times 100=\frac{\mathrm{ME}\times \mathrm{RE}}{100}$$

where *A* represents the average peak area of the standard solution (*n* = 5), *B* represents the average peak area of a post-extraction spiked sample (*n* = 5) and *C* corresponds to the peak area of the standard spiked before extraction (Figure 3). When ME (%) > 100%, ion enhancement is observed, while when ME (%) < 100%, ion suppression occurred. Through this approach, the authors demonstrated that ME (%), RE (%), and PE (%) are closely correlated. They suggest the application of the methodology at three different levels of concentration (low, medium and high) by using quality control samples. The advantage of this procedure is that recovery and MEs are assessed together, and can be combined with accuracy and precision studies, minimizing the number of experiments to perform and obtaining information of MEs among the whole calibration range.

The results obtained with Equation (1) can be termed “absolute” MEs, since it considers only the differences in response of standards prepared in neat solvent compared to the presence of the matrix. The comparison of “absolute” MEs between different lots or sources of sample evaluates the “relative” MEs extension, which is another important parameter during the development of a new analytical method.

Heller [52] presented another approach, “matrix effect maps,” for visualizing the impact of various parameters on matrix effects associated with a given method. Matrix effects were studied as a function of the amount of co-injected matrix extract. In spite of its potential, this method has been applied only limitedly.

At the end of this preliminary MEs evaluation, besides the ME (%) value, we have important information about the best combination of extraction and chromatographic processes, which includes both matrix effects and extraction efficiency on each analyte at the same time.

### 2.3. Slope Ratio Analysis

Sulyok and co-workers [28] developed a method in LC/MS-MS for the semi-quantitative screening of 87 mycotoxins in moldy food samples. They evaluated the method performances by preparing spiked samples, mix standards in neat solvent and matrix-matched calibration standards at different calibration levels.

For the spiked sample preparation, a model matrix was used. Each of them followed the sample preparation procedure that consisted in a dilution 1:1 with solvent. After injection into the LC/MS-MS system, three calibration curves were obtained. The corresponding slope ratios were used to calculate the apparent recovery (R_A_), the signal suppression-enhancement (SSE) due to MEs and the overall recovery percentage including the extraction step (R_E_), as according to Equations (5)–(7) respectively:

(5) $$\mathrm{SSE}\left(\%\right)=100\times slope_{matrix-matched\;standard}/slope_{liquid\;standards}$$

(6) $$R_{E}\left(\%\right)=100\times slope_{spiked\;sample}/slope_{matrix-matched\;standard}$$

(7) $$R_{A}\left(\%\right)=100\times slope_{spiked\;sample}/slope_{liquid\;standards}$$

This modified approach evaluates the same parameters obtained by Matuszewki et al. [26] in an entire selected range of concentrations instead of a single level.

### 2.4. Evaluation of Relative MEs

In addition to “absolute” MEs, analysts must take into account the possible occurrence of “relative” MEs, that represents a sample to sample variability of the matrix [26], the importance of which is also proven by the fact that the FDA requires the assessment of “relative” matrix effect in bioanalytical methods [45].

Evaluation of relative MEs becomes critical when matrix-matched calibration or background subtraction calibration approaches are used to compensate for MEs. Typical examples of “relative” MEs are encountered during the analysis of such biological fluids as urine or blood, in which the presence and concentration of sample interferences depend on factors such as patient variability, time points (hours, days, weeks) after dosing, diet, renal function, and metabolism [3,46,47].

In addition, relative MEs have been reported for other matrices such as vegetables, and in particular during analysis of pesticides in fruits and vegetables from different sources, for example, those grown in different regions [48].

In general, most authors do not discriminate between these two phenomena, and the lot variability is faced with the “classic,” approach used for most general absolute MEs, that is, to overcome MEs by taking care in sample preparation and pre-treatment, chromatographic conditions, and mass-spectrometric sources.

Interestingly, Matuszewski [46] achieved a good quantitative indicator of the presence/absence of relative MEs from five different lots of biofluid. The author reported that if the coefficient of variation (CV%) of their standard line slopes does not exceed 3–4%, the relative MEs can be considered negligible, and concluded that the comparison of the CV% values of standard line slopes of these five different lots with the analogous values obtained by repeated analysis (*n* = 5) in a single lot may also serve as an excellent measure of relative MEs.

On the contrary the recent FDA and European Medicines Agency (EMA) guidelines, for validation of bioanalytical methods [45,49], report the necessity to process six lots of biofluids at two levels of concentration (close to the LOQ and the ULOQ), admitting a CV% not exceeding 15%.

## 3. Compensating for MEs

When sensitivity is reached, efforts to overcome MEs can focus on compensating for them by using the suitable calibration approach. The convenient calibration method is strictly dependent on the availability of a blank matrix, defined according to the most recent FDA Guidelines as “*a substance that closely matches the samples being analyzed with regard to matrix components*” [53]. A blank matrix affords the possibility to estimate the background level of analyte(s), to verify the sample matrix, and to use equipment that does not interfere with or affect the analytical signal [54].

When the blank matrix is available (Figure 1), it can be spiked with a known amount of analyte, and the matrix effect can be assessed by the post-extraction spike method proposed by Matuszewki et al. [26] with a quantitative estimation (Figure 2A). In addition, all the other validation parameters like recovery, accuracy, precision, linearity, sensitivity, and the preparation of low, medium and high concentration quality control samples can be assessed [54]. The data can also be used to evaluate robustness of the method resulting from changes in the sample matrix.

According to the FDA definition, when no blank matrix is available, it may be impossible to find a matrix similar to the sample without the target analytes, because they are ubiquitous substances, such as hormones [55] in plasma or serum. But in practice, a blank matrix may be unavailable simply because quantities are limited, for example in the case of cerebrospinal fluid (CSF) [56] or previous samples.

### 3.1. Case Study I: The Blank Matrix Is Available

When a blank matrix is available, the matrix-matched calibration method can be performed [57] by post-extraction spiking of a representative blank matrix with increasing amount of the analytes. The representativeness of the blank matrix is generally obtained using a pool of blank matrix coming from different lots in order to compensate as much as possible for the sample to sample variability of the matrix (relative MEs). We recommend using five different lots (*n* = 5), in line with the proposed methodology for the evaluation of the relative MEs [46]. In addition to verifying linearity response in the calibration range, this calibration approach provides information related to the sensitivity of the method, when the lowest calibration level corresponds to the limit of quantitation. Moreover, each calibration level has to be injected repeatedly (*n* = 5), providing information about the precision of the method. This very effective calibration approach is the most common method for overcoming ME when a blank matrix is available, even if large amount of blank matrix is required for processing and its use contributes to the contamination of the MS system in the same way as the sample. This is a particular concern for bioanalytical analysis, especially for short analytical run where the number of calibration standard and unknown samples are comparable. Furthermore, a matrix-matched calibration strategy cannot totally compensate for MEs. In those cases, the accuracy of quantitation can be improved by using stable isotope-labelled internal standards (SIL-ISs), when they are commercially available [58,59,60,61].

The literature reports the importance of employing the SIL-IS in which the isotope used for labelling provides the best MEs compensation (Table 2) [62]. In fact, the ability to compensate for MEs is strictly related to the perfect coelution between the analyte and the correspondent SIL-IS. In chromatography, the retention times are related to the physicochemical properties of the substance, and coelution between the target molecule and its labelled analogue depends on the stable isotope used. The isotopes used for this purpose are ^2^H, ^13^C, ^15^N and ^18^O. There is a greater difference in physicochemical properties between hydrogen isotopes and isotopes of the other elements. During the chromatographic runs, co-elution between ^13^C, ^15^N and ^18^O labelled internal standard and the correspondent unlabeled analytes was better than that obtained with ^2^H labelled internal standards. In addition, the chromatographic resolution between the analyte and its ^2^H labelled analogue increased with the number of ^2^H substitutes, resulting in higher isotope effect in the chromatographic separation.

For these reasons, research studies on the differences between the use of ^2^H- and ^13^C-labelled ISs concluded that ^13^C is the isotope of choice, as it corrects up to 70–80% of MEs and it results in an improved ruggedness. In fact, according to their similar behavior, SIL-IS and the correspondent unlabeled analyte will be affected in the same way by the deliberate variations of the method parameters that are used to investigate the method ruggedness [63]. Then, the ratio between the two species in MS signal response will be unaffected.

In addition, Hewavitharana [64] reflected on the uselessness of matrix matching of the calibration standards when an SIL-ISs are used: although the magnitude of the individual responses of analyte and internal standard will be affected by MEs, the ratio of responses will be unaffected. More in detail, the matrix matching calibration approach is commonly used to overcome MEs, but its effectiveness is strictly related to the availability of a blank matrix and how much the blank matrix is representative of each real sample. Instead, the SIL-IS mimics perfectly the response of the corresponding unlabeled analyte, independently from the dissolving system (pure solvent or matrix). This means that whether the calibration standards are prepared in real matrix or in solvent, the calibration curve, in terms of ratio of analyte response/internal standard response vs. ratio of analyte concentration/internal standard concentration or analyte concentration, will be exactly the same [65]. The use of SIL-ISs is particularly useful when a blank matrix is unavailable.

Should the obtained results prove unacceptable in terms of accuracy and precision, exceeding relative standard deviation percentage (RSD%) of 15% (20% at the LOQ level) [45], a bland preparative step can be performed in order to decrease the concentration of interferences in the matrix. In this case, the strategy to minimize MEs has to be planned in order to obtain the maximum efficiency in terms of cleaner samples, also taking into consideration the time consumed in the preparative step.

### 3.2. Case Study II: The Blank Matrix Is Not Available

When the blank matrix is not available (Figure 1) (Table 3), it is not possible to conduct matrix-matched calibration, and thus the only way to correctly evaluate MEs is by using the post-extraction addition proposed by Matuszewky [26], because the evaluation of the method ruggedness became a crucial point. Relative MEs has to be assessed by analyzing different batches of the matrix or pooled matrix and examining the resulting reproducibility of the process. In fact, the lack of blank matrices makes it is necessary to subtract the background response of the analytes from the response of the added standards [66], except when the standard addition method is performed. Thus, different background levels could make MEs evaluation irreproducible over time or between different laboratories.

Even when the blank matrix is not available, it is possible to compensate for MEs by choosing a calibration method that is effective and not time-consuming, but in this case, a calibration method other than matrix-matched calibration has to be used. A recent comprehensive review by Thakare et al. [23] evaluated four approaches: the standard addition, background subtraction, surrogate matrix and surrogate analyte methods.

The standard addition method requires the same extract to be spiked with the analyte at different concentration levels in order to construct a calibration curve for each sample. Using this approach, the matrix variability is not a crucial point and the relative MEs need not be assessed. Thus, this method is very effective, giving good results even when relative MEs phenomena are observed, or when a coelution between the analyte and an interference responding to the same MS signal is observed. However, it requires a large amount of sample and is also very time-consuming, because spiked samples must be run for each unknown [67,68]. Thus, the number of injections needed corresponds to the number of samples multiplied by the number of calibration levels performed (at least two injections for each sample, up to four, when it is performed single to three points of standard addition respectively). For this reason, this calibration approach is unsuitable for routine analysis.

The building of a calibration curve by subtracting the background level has the same drawbacks in terms of the low reproducibility reported previously for MEs evaluation, as it is strictly related to the variability of the matrix or pooled matrix used in the analyses, especially when particular sensitivity is required and the background concentration level of the analytes is high and variable. This results in high LOQs of the method [69,70].

The use of surrogate matrix could be a useful approach, especially for such biological matrices as urine [71,72,73], serum [55,70], and cerebrospinal fluid [74]. Surrogate matrices reported in the literature are neat solvent, stripped and artificial matrices. They act in a similar way to a blank-like matrix and are used for the assessment of the analytical method performance. A stripped matrix is obtained by proper treatment of the real matrix (the use of activated-charcoal as an absorbent of the target analytes is the most common approach) [75,76,77,78,79,80,81,82,83,84,85,86], but an artificial matrix is prepared in order to reproduce the authentic matrix, except for the analytes, in terms of composition, analyte behavior and salt content [87,88,89,90,91].

In addition, neat solutions such as water, methanol, water/methanol/acetonitrile, or 0.1% HCl can be used as a surrogate matrix [92,93,94,95,96,97,98,99]. When a surrogate matrix is used, it is necessary to demonstrate that this approach does not affect quantitation in the entire concentration range.

Finally, the authors describe the surrogate analyte method, identifying the SIL-ISs, previously described, as the surrogate standards. The possibility to use SIL-ISs for quantification of the correspondent unlabeled analyte has to be verified and the response factor (ratio unlabeled/labeled analyte) has to be close to unity in the entire calibration range. Otherwise, the response factor must be incorporated into the regression equation used for calibration.

Many studies have reported on the effectiveness of compensating for MEs by using SIL-IS, because of its similarity to the target analyte and the absence of endogenous background [64]. Despite the importance of SIL-ISs in quantitative analyses in liquid chromatography-mass spectrometry techniques, their limited commercial availability and cost shrink applications in routine laboratory analyses. When SIL-ISs are added before the extraction step, they are able to correct all random errors occurring during both the preparative step and instrumental analysis, thus improving method ruggedness, precision, and accuracy.

Sometimes, research laboratories produce their own SIL-ISs suitable for specific topics, doing their own chemical synthesis [72] or producing them by growing organisms on labelled feed, such as yeast grown on medium containing ^13^C-labeled glucose for quantitation of NAD metabolite in cellular extract [100]. According to the last approach a ^13^C yeast extract was obtained, and MEs was evaluated by spiking the fully-labelled extract with an unlabeled analyte mix standard thanks to the lack of endogenous interferences. From this point of view, the fully-labelled extract is complying with the surrogate matrix definition and can be used for the same purpose: calibration curve construction, MEs evaluation, precision, accuracy and sensitivity estimations.

When efforts to compensate for MEs with a suitable calibration approach do not give accurate or precise results, it is necessary to decrease the interaction of the target analytes with the others matrix components. Several strategies to reach this goal are reported in the literature, such as simple matrix dilution, the choice of the most suitable interface-system, reduction of co-elution by the chromatographic system and at least the physical removal of the interfering species by an extensive clean-up step. The right approach should be chosen on the basis of the maximum efficacy and the shortest time required.

## 4. Minimizing Matrix Effects

Minimizing MEs, and not just compensating for them, is the correct approach in two cases. The first case is when sensitivity is a critical parameter, and thus a pre-concentration step is needed. This may also afford a cleaner extract sample, especially by using specific extraction techniques able to reduce the interference concentration. However, sometimes MEs may increase when the pre-concentration step is performed, due to the concentration of both the target analyte and the interference species in the final extract, resulting in poor sensitivity gain. The necessity of reducing MEs by performing extensive clean-up also occurs when the calibration approach used does not compensate adequately for the MEs. In this case as well, an extensive clean-up step should be performed.

### 4.1. Sample Dilution

MEs can be reduced by two simple approaches, namely, sample dilution and smaller injection volumes. In both cases, the quantity of matrix components introduced into the analytical system is lower, resulting in reduced matrix suppression and the possibility of using standards in neat solvent as a calibration method [30].

Most frequently, MEs can be reduced by sample dilution [101], but this approach is appropriate only if method sensitivity is preserved [35]. For example, in one study a selective and speedy LC-MS/MS method was developed to determine six trichothecene mycotoxins in rice medium. The analytes were extracted from the rice medium and diluted with acetonitrile/water (85/15, *v*/*v*) in order to minimize the effects of matrices [102]. Diluted solutions were analyzed by LC–MS/MS with electrospray ionization (ESI) interface in negative or positive ion mode and the multiple reaction monitoring mode. Recovery rates were 76–106% with a spiked level at 1–6 μg/kg of mycotoxins, which corresponded to the limit of quantitation.

In another study, Stahnke et al. [103] investigated the relationship between matrix concentration and MEs, in particular, ion suppression of electrospray ionization, in a series of pesticides present in Quick, Easy, Cheap, Effective, Rugged and Safe (QuEChERS) extracts. Examining 10 dilution levels from undiluted up to 1000-fold, the authors found a logarithmic correlation between MEs and dilution factor. Specifically, they demonstrated that dilution by a factor of 25–40 reduces ion suppression to less than 20% when the initial suppression was ≤80%.

Ferrer et al. [104] demonstrated that sample dilution may be considered an easy and effective method to reduce MEs. They evaluated MEs of 53 pesticides in several vegetable matrixes under dilution, finding that a dilution factor of 15 proved sufficient to minimize MEs in most cases. For the more problematic pesticides, the use of SIL-ISs was suggested as a possible solution.

Kruve and co-workers [105] investigated MEs as a function of dilution for a series of pesticides in five vegetal matrixes. Since it is generally demonstrated that dilution can eliminate MEs, but in some cases only reduce it, the authors proposed a new extrapolative dilution approach that proved highly accurate compared to the simple sample dilution. In this study, information on whether and how MEs are reduced with dilution was obtained by plotting the calculated concentration of analyte in sample against the dilution factor. Actually, the authors observed three different situations: (a) absence of MEs (b) suppression of MEs by dilution, and (c) MEs not fully eliminated by dilution.

The LC–MS/MS “dilute and shoot” method was applied for the determination of 295 fungal and bacterial metabolites. The MEs were dependent on the type of food matrix: when the MEs were at the lowest level, 59% of analytes were not influenced by ion enhancement or suppression, while when MEs were at the highest level only 10% of analytes did not suffer from signal suppression or enhancement [106]. Application of dilute-and-shoot LC-MS can be considered possible for substances with low required detection levels or limited ionization efficiency, because of the progressive increase in sensitivity of modern instruments [107].

Quantification of itraconazole was possible by using only 10 μL of whole blood: the itraconazole was slightly affected by the matrix (91.2%), whereas there were slightly positive MEs observed for hydroxy-itraconazole (110.7%) [108]. When the sample dilution approach or injection of smaller volumes does not provide adequate solutions for overcoming MEs, sample preparation becomes compulsory to selectively eliminate or reduce co-eluting interferences.

### 4.2. Mass Spectrometric Conditions

The first and simplest approach to overcome MEs for an analyte is to adjust the mass spectrometric conditions. By keeping the preparation and/or chromatographic analytical procedure unmodified, it is possible to limit the MEs by simply modifying the MS conditions. Any adjustments serve to detect the best analyte signal with respect to the lowest background ionization.

One of the first parameters to be considered is ionization polarity, which is chosen according to the chemical structure of the analyte and the mobile phase. Several studies showed that negative ionization was less susceptible to MEs because the number of the matrix component giving response in the negative ion mode is lower than in the positive mode [35,48]. Thus, when it is applicable, the negative ion mode should be preferred to the positive one.

Experimental findings indicate that the extent to which different ionization sources for HPLC are susceptible to MEs varies according to the particular ionization/evaporation processes occurring inside each interface, including all the atmospheric pressure sources, namely electrospray ionization (ESI), atmospheric pressure chemical ionization (APCI), atmospheric pressure photo-ionization (APPI), direct-electron ionization (direct-EI) [109], and direct analysis in real time (DART) [110]. In this review, according to give a practical guideline in overcoming MEs, only the commercially available API sources will be discussed.

Among the atmospheric pressure ionization (API) techniques, ESI is the most widely used source, even though it is also the one most affected by MEs [10], because of the several steps involved in the gas-phase (GP) ion formation: charged droplet formation, addition of the charge to the analyte in the liquid phase (LP), solvent evaporation and droplet fission, creation of the GP ion through the charge residue model (CRM) or ion-evaporation model and sampling of the GP ion. Any occurrence that decreases the rate of one of these steps may be detrimental to the transfer of the ion generated in LP to GP and consequently create signal suppression. In addition, the design of the source may affect the effectiveness of the process, contributing to MEs [37,111,112]. Today the ESI sources commercially available can have three different geometries: the off-axis, where the spray is positioned 30–45° relative to the *x*-axis between sampling capillary and the first quadrupole (e.g., Ion Spray and Turbo Ion Spray interfaces); the orthogonal geometry where the relative angle is 90° (e.g., Jet Stream Source from Agilent and Turbo V™ from AB Sciex) and Z-spray geometry which present a double orthogonal sampling. Orthogonal and Z-spray geometries usually enhance sensitivity by preventing the orifice from clogging with non-volatile materials. In addition, the Z-spray configuration is able to efficiently separate neutral molecules and solvent vapor from the ion stream to the spectrometer. More in detail, Ghosh et al. [37] reported a comparison between the most available sensitive orthogonal and Z-spray geometries in monitoring the ionization of phospholipids, that are considered the major interferences in bioanalytical analysis performed by using ESI interface. Even if use of the orthogonal geometry produced fewer MEs than did that of a Z-spray configuration, they offered no general or conclusive assessment on which one is less prone to MEs. The most important observation they reported is that different phospholipids were ionized when different ion source designs were used. This phenomenon confirmed the role of the interface configuration on MEs and the necessity to evaluate case by case to ascertain which one is the best. The same conclusion was reported by Stahnke et al. [111], who observed that the spray geometry of an ESI source had no significant influence on the extent of MEs. Nevertheless, they underlined that the use of a more sensitive ion source offers the possibility of injecting more diluted samples or smaller injection volumes, which may reduce MEs.

Instead, in the APCI source, the analyte passes into the GP as a neutral molecule and only subsequently does the ionization process occur by chemical ionization [113]. In this way, the use of APCI avoids all the suppression mechanisms present in the LP. In fact, several studies have shown that APCI-MS is less affected by ME than ESI-MS [3,4,46,48,114,115,116,117], even though MEs are not infrequent with APCI [3,48,118,119], probably due to the co-precipitation of analyte with non-volatile matrix compounds in the LP negatively affecting the evaporation of the analyte as a neutral molecule [12], or differences in electron affinity between components of the GP [120].

In APPI, the neutral molecules in the GP are exposed to ultraviolet light from a krypton lamp. The photons emitted from this lamp have a specific energy level (10 electron volts, or eV) that is just right for this process: it is high enough to ionize the target molecules, but not so high as to ionize air and other unwanted molecules. This technique is able to ionize non-polar or low polar compounds that are not efficiently ionized by the other API sources. Even if its applications are still limited and the MEs associated with APPI have not been investigated thoroughly, the results reported in the literature indicate that it affords generally lower suppression phenomena than ESI and APCI, due to the fact that the process is not based on proton affinity [121,122].

### 4.3. Chromatographic Conditions

The use of appropriate chromatographic conditions helps to improve the separation of the analyte from interfering substances, avoiding its co-elution with matrix [10,39,40,48,123]. The selection of the most appropriate elution conditions (mobile phase and stationary phase) may be considered the first and simplest approach to separate the analyte under investigation and thus suppress MEs [124].

Since the regions more frequently affected by interferences are the solvent front, where high polar and unretained compounds are eluted, and the end of the chromatographic gradient, where the most retained substances are eluted, it is preferable to adjust the analyte retention time in order to be far from these two areas [10]. This may be achieved by appropriately selecting the mobile phase, which of course takes into account the solubility of the analyte. In the case of ionizable analytes, it is also preferable to adjust the pH of the mobile phase in order to modify the retention time of eluting substances [104,125,126]. The pH value could also affect sensitivity; Chambers et al. [125] reported higher sensitivity for basic analytes at high pH values. The proposed reasons stand on the neutral form of the analytes at high pH values and the correspondent elution at higher retention time and higher percentage of the organic modifier, that corresponds to higher desolvatation efficiency in the ESI source. Mobile phase additives may influence ME as well [48,127,128]. The mobile phase may be delivered in the isocratic mode (constant composition) [37,126,129,130], and in gradient elution mode [2,36,104,126,131,132], helping to reduce the matrix effect.

In addition to the possibility of modifying the mobile phase to overcome MEs, the choice of a more selective stationary phase offers a supplementary solution. Hydrophilic-interaction liquid chromatography (HILIC) offers advantages over reverse phase chromatography (RPLC), particularly for highly polar compounds that are slightly retained in RPLC eluting in the first part of the chromatogram, which is one of the regions more affected by MEs [133,134]. HILIC mode uses a polar stationary phase and an aqueous-polar organic mobile phase containing a high percentage of the organic component (exceeding 60%). The organic modifier mostly used is acetonitrile, providing low backpressure thanks to its weak viscosity. In addition, high acetonitrile content assists the formation of smaller droplet in ESI source and enhances desolvation efficiency by decreasing the number of evaporation cycles. Thus, the use of an HILIC system provide sensitivity enhancement and a reduction of MEs with respect to RPLC, where polar compounds are eluted with highly aqueous mobile phases [135,136].

The advantages of HILIC versus RPLC mode are more evident when old-generation ESI devices are used. In fact, the modern ESI sources are able to efficiently desolvate highly aqueous mobile phases, even at high flow rate. Recently, the role played by the design of the ESI source on sensitivities obtained in HILIC-MS and RPLC-MS was studied [112]. Between the recent orthogonal sources such as AB Sciex Turbo V™ and Agilent Jet Stream, the first provided limited improvement of sensitivity by using HILIC rather than RPLC. Otherwise, the latter showed strong influence of the mobile phase flow rate and composition on the overall measured sensitivity. In addition, compared to the AB Sciex Turbo V™, the Z-spray source design of Waters Xevo TQ-S seems more adapted to HILIC than does the RPLC mode, especially at high flow rate.

The HILIC-MS/MS method has been widely applied for the quantitative determinations of different drugs in human plasma [137,138,139,140,141,142] because it requires only a small volume of plasma, thanks to the improved sensitivity and rapid sample pretreatment, by-passing the evaporation of the organic solvent and reconstitution of the sample in a highly aqueous solvent, when the protein precipitation procedure or the Solid Phase Extraction step are performed, unlike in RPLC mode. These advantages permit routine application of the HILIC method to bioanalytical analyses [143].

Mess et al. [144] evaluated retention times and MEs associated with phospholipids from human plasma extracts under HILIC conditions. The authors observed that phosphatidylcholine and lysophosphatidylcholine phospholipid retention times varied greatly between columns operated in different HILIC conditions. Therefore, MEs associated with phospholipids could present a quantitation problem if not evaluated thoroughly during method development.

In general, HPLC may show several limitations concerning separation power, depending on time available for analysis, as well as on instrumentation performance (pressure pump, column temperature, properties on the packing material) and the thermal stability of sample and packing material [46].

Ultra-high-performance liquid chromatography (UHPLC) is another effective tool in overcoming MEs. It is characterized by higher speed, resolution, and sensitivity than normal HPLC [145,146,147,148,149,150]. Despite its high chromatographic performance, the UHPLC has a limit in terms of peak capacity, which, however, can be overcome by developing two-dimensional liquid chromatography techniques (LCxLC, (2D) method) that can resolve samples that current one-dimensional liquid chromatography methods are unable to resolve [151]. In particular, 2D separation makes it possible to improve peak capacity (maximum number of resolvable peaks) as defined by Guiochon et al. [152].

However, it is important to develop appropriate method through a rigorous selection of such chromatographic parameters as stationary and mobile phases, column formats, and chromatographic conditions [151]. One of the most important parameters to be assessed is the orthogonality of single and coupled columns, necessary for the determination of the optimal column combination [153].

Česla et al. [154] reported that the 2D LC-MEKC method offers high orthogonality and peak capacity for the separation of complex samples, but unsatisfactory sensitivity, due to the injection of only small fractions collected from the first LC dimension into a capillary for the second-dimension MEKC separation. However, with certain experimental conditions and precautions, such as the dilution of the first column effluent with weak solvent prior to injection into the second-dimension column, the sensitivity of this technique can be preserved [155,156].

### 4.4. Clean-Up Optimization

Sample pre-treatment procedures are applied to reduce the amount of matrix components that are introduced into the analytical system. They may involve a more selective analyte extraction procedure or a more extensive sample clean-up prior to injection into the LC-MS system [46,157,158].

#### 4.4.1. Removal of Proteins

In presence of biological matrixes, and thus in presence of proteins, the simplest and fastest method for preparing samples is protein precipitation [37,40,134,159,160,161] (Table 4). Common methods for protein precipitation include salting out and precipitation with organic solvents: precipitation with acetonitrile was shown to be a better choice as organic solvent than methanol for PPT [125,129,136,162,163]. Unfortunately, this method fails to fully remove other endogenous compounds generally present in biological samples, such as lipids, phospholipids, and fatty acids. Consequently, it is responsible for ion suppression in ESI [3,12,125,164,165,166].

In addition, precipitation of proteins with acids catalyzes the hydrolysis of several conjugates such as glucuronides and sulphates [167,168], giving rise to a necessary neutralization step before injection. Alternatively to an organic solvent, the use of a ZnSO_4_ solution is reported to be an effective method for PPT [169,170]. According to the literature, this system is also useful for decreasing the amount of phospholipids in the sample, with great advantages in overcoming MEs.

#### 4.4.2. Phospholipid Removal

Phospholipids (PLs), a class of lipids with peculiar characteristics, are the main components of cell membranes and may be found in different concentration levels in many biological matrices, such as plasma, urine, tears, cerebrospinal fluid, synovial fluids and in tissues, even if the most affected are plasma and serum samples [173,174]. They can be divided into two classes: glycerolphospholipids and sphingomyelins (SM) [175,176].

Glycerolphospholipids are composed of several sub-classes, which are phosphatidic acid (PA), phosphatidylcholine (PC), phosphatidylethanolamine (PE), phosphatidylglycerol (PG), phosphatidylinositol (PI), and phosphatidylserine (PS). They are able to suppress ionization of neutral, basic and acidic analytes in both the positive and negative ionization mode [177].

The first step when dealing with these types of matrices is to qualitatively investigate the MEs caused by the presence of phospholipids (Table 5). Little et al. [169] reported a well-received technique based on monitoring the selected transition 184—184 in positive ion mode using ESI source. PC, lysoPC and SM can be monitored using this technique. More recently, two other approaches have been proposed in order to detect all classes of plasma PLs [177]. The first, recommended for a qualitative assessment, is based on comprehensive precursor ion/neutral loss scans involving positive ESI precursor ion scan of *m*/*z* 184, positive neutral loss scan of 141 Da and negative precursor ion scan of *m*/*z* 153. The second technique is based on using class-specific SRM transitions to monitor phospholipids selected to represent every class of phospholipid, achieving a complete and quantitative assessment of PLs during the development and application of LC-MS bioanalytical methods. Preliminary monitoring of PL elution behavior during the cycle can give important information on what modifications to the settings of the chromatographic conditions need to be done in order to have the analyte elute at a separate point. Unfortunately, not all the PLs are eluted off the column, giving potentially irreproducible results when high sample volumes are used. Many strategies to remove PLs are offered in the literature. The simplest method is PPT, through the use of acids, bases or organic solvent, but this strategy removes proteins without extensively removing PLs, as previously reported. Chambers et al. [125] provided a systematic approach to optimize sample preparation in bioanalytical LC/MS/MS assays in order to minimize MEs due to the presence of endogenous phospholipids in plasma. The authors compared several sample preparation methods, such as protein precipitation (PPT), liquid–liquid extraction (LLE), pure cation exchange solid-phase extraction (SPE), reversed-phase SPE, and mixed-mode SPE.

PPT proved the least effective sample preparation technique, causing significant ion suppression for many analytes, because of the presence of many residual matrix components. Reversed-phase and pure cation exchange SPE methods resulted in cleaner extracts and reduced MEs compared to PPT. The most efficient method for reducing phospholipid levels was the polymeric mixed-mode strong cation exchange SPE, because it combines the retention mechanisms of reverse-phase and ion exchange. LLE also provided clean final extracts, even if analyte recovery was unacceptable, particularly for polar analytes.

Jiang et al. [165] compared results from two different sample preparation methods, PPT and the Solid Supported Liquid Extraction (SLE), a process that is similar to LLE in terms of mechanics, but requires less solvent, involves the use of inert and porous material over which the aqueous sample is poured, and which adsorbs the sample, as well as performance of extraction with an organic and immiscible solvent. They systematically evaluated the efficacy of SLE in reducing MEs for 10 model pharmaceutical compounds of different physicochemical properties in LC-ESI-MS/MS. The MEs were considerably reduced for all the analytes under SLE in comparison with PPT, because the former was able to remove the majority of phospholipids when appropriate loading buffers and eluting solvents were applied.

An advanced technique called HybridSPE-PPT (Sigma Aldrich, St. Louis, MO, USA), which combines the simplicity of precipitation with the selectivity of SPE, was proposed to overcome limitations associated with PPT. This new technique is able to minimize MEs caused by the presence of phospholipids and proteins in biological samples [130,178]. This approach has been commercialized by several companies, creating products specific to this task: HybridSPE™, the system from Sigma Aldrich, reduces the levels of residual phospholipids in biological samples, leading to significant reduction in MEs, and allowing the recovery of much cleaner extracts than those obtained with conventional procedures [179].The same goal is achieved by using Ostro™ from Waters [180], which has the same performance of the Phenomenex Phree™ technology [181] and the Captiva™ system from Agilent [183].

Recently, a new and effective approach to removing both proteins and phospholipids was proposed by Salatti-Dorado et al. [171]. It is based on the dual precipitation of both species by using restricted access, volatile supramolecular solvents (RAM-VOL-SUPRAS), nanostructured liquids produced by colloidal solution of amphiphiles by spontaneous processes of self-assembly and coacervation. Salatti-Dorado and co-workers applied this system to the extraction of bis-phenol A from urine samples. The RAM-VOL-SUPRAS system was obtained adding the hydrolyzed sample to a solution of hexanol in THF. The SUPRAS was formed by coacervation of hexanol in the presence of urinary water. Protein precipitation was immediately performed and proteins were separated by centrifuge. An aliquot of the SUPRAS extract was evaporated to dryness. The residue containing the PLs precipitate was treated with a mixture 50:50 of methanol:water to selectively dissolve bis-phenol A, eliminating or dramatically reducing MEs. In fact, the PL precipitations achieved by evaporation were re-extracted in a minimal amount thanks to slower diffusion of proteins on the extraction solvent compared to that of the analyte. The proposed system is expected to afford high extraction efficiency for analytes in a wide range of polarities as a result of the large number of binding sites and different polarity regions present in the nanostructure making up the SUPRASs.

Similar results in terms of decreasing MEs were obtained by using a Parallel Artificial Liquid Membrane Extraction (PALME) technique for quantification of non-polar basic and non-polar acid drugs in human plasma [182]. The PALME system involves an acceptor plate placed on the top, and a donor plate on the bottom. The acceptor plate contains 96 flat membranes which are impregnated with an organic solvent, while the donor plate contains the sample, creating a three phase sandwich-like system. The plates are clamped together and the whole assembly is agitated on a platform. The analytes are extracted during agitation using the pH as driving force. In fact, the pH is adjusted to ensure uncharged analytes in the sample and charged analytes in the acceptor solution. The results revealed a complete absence of ion suppression and high recovery percentage, indicating that the PALME system has great potential for future applications.

#### 4.4.3. Removal of Lipids

Lipids are interfering species when an LC-MS method is performed on both biological and food matrices. The efficient removal of lipids from the extract prior to instrumental analysis is crucial to limit interference and increase sensitivity and reproducibility of the analytical method (Table 6). Simple and common defatting procedures reported in the literature are LLE with hexane [184,185] and freezing lipid precipitation (LFP) [186].

The latter is an effective approach especially for samples with high lipid content. The FLP procedure is performed by freezing the sample at −24 °C in a freezer, after which the frozen lipids can be easily removed by filtration. This approach was demonstrated to be an effective sample clean-up, and in addition it can be easily combined with a subsequent SPE clean-up step, if necessary.

Instead of using low temperatures, the co-precipitation of fat and proteins was achieved by Rodríguez-Gómez et al. [187] by using a solution containing zinc and tungsten salts in an acidic media, the same used for milk treatment in the analysis of lactulose by the International Dairy Federation [188]. They obtained the formation of a solid white precipitate that was easily separated from the solution that contained the target analyte, thus achieving good performance in terms of sensitivity.

Another common and easy procedure for defatting consists in using a combination of two solid sorbents such as primary secondary amine (PSA) and C18 in a dispersive solid phase extraction (d-SPE) mode [189]. It corresponds to the clean-up step of the QuEChERS extraction technique. The C18 sorbent is specific for the removal of co-extracted fat and other lipophilic compounds from the acetonitrile extracts. Similar approach was applied in another study with the intent to achieve negligeable matrix effect for mycotoxins determined in peanut, pistachio and almond [190]. Zirconium dioxide-based sorbents were also shown to be very effective in reducing MEs in fatty matrix. They are generally used in combination with PSA instead of the C18 sorbent [191]. Baduel and co-workers [172] explored the efficacy of the Zirconium dioxide-based sorbents as dispersive solid-phase extraction (d-SPE) and protein-lipid removal filter cartridges (Captiva ND Lipids) for the clean-up of lipids, proteins and other impurities present in biological matrices. They concluded that Captiva ND Lipids cartridges are the best option for reducing MEs in LC-MS applications.

#### 4.4.4. Removal of Sugars

Sugars are common interference species mostly present in food matrices. Since the sugar level of honey exceeds 75%, it could be considered a probe matrix for evaluating the most efficient technique for overcoming MEs related to the presence of sugars. In the literature, the most common (actually, the only) approach consists in using a solid adsorbent such as PSA. However, this procedure is not selective, as it removes sugars along with other polar interferences [192,193,194]. This approach provides generally good results in terms of overcoming of MEs.

## 5. Conclusions

Despite its versatility, LC-MS has its Achilles heel in the extensive MEs that occur in the interface system due to the interaction of the analyte with the other components of the matrix that act as interference species. These phenomena lead to unpredictable and variable ionization suppression or enhancement, affecting method performance in terms of precision, accuracy, ruggedness and sensitivity. When sensitivity is not a crucial parameter, the analyst may focus exclusively on compensating for the MEs, using the proper calibration strategy. The choice is strictly related to the availability of a representative blank matrix. When it is available, a matrix-matched calibration procedure can be used, which allows efficient correction of MEs, even if this calibration method is not able to completely compensate for MEs when great sample to sample variability MEs is observed (relative MEs). The accuracy and ruggedness of the analytical method can be further improved by the use of internal standards. The most effective internal standard in terms of compensation for MEs are the SIL-ISs, acting as surrogate analytes. When the blank matrix is not available, other calibration methodologies than matrix-matched calibration have to be used, such as the background subtraction, standard addition, surrogate matrix and surrogate analyte methods. Background subtraction has the same limitation of the matrix-matched calibration method regarding the compensation for MEs when high values of relative MEs are observed. In both cases, relative MEs are a critical parameter during the validation process, one that must be evaluated and quantified. When a surrogate matrix approach is used, it is necessary to confirm that the surrogate matrix behaves like the real matrix. Similarly, when the surrogate analyte calibration method is performed, it is necessary that the surrogate analyte respond to the MS signal in the same way as the target analyte. When SIL-ISs are used as surrogate analytes, the ratio between the analyte and the SIL-ISs has to be equal to the unity and it has to be constant over the entire calibration range, otherwise a correction factor must be inserted into the calibration curve equation. Unfortunately, SIL-IS are not always commercially available and may be expensive. When the available calibration strategy does not completely compensate for MEs, or the method lacks in sensitivity, it is crucial to take measures to reduce or eliminate MEs. Several strategies for this purpose are reported in the literature. Case by case, the easiest and fastest approach has to be chosen. The dilution approach has proven to be effective, easy to perform, and fast when it does not badly affect the necessary sensitivity of the method. Other generally used strategies with a proportional increase in the time required are the identification of the most suitable MS conditions, of the optimal chromatographic separation and of the most effective clean-up step. Useful techniques for evaluating and comparing the different strategies and choosing the best one are post-column infusion, applicable when a blank matrix or a surrogate matrix are available, even if it can give only qualitative information on MEs, or post-extraction addition, which is applicable independently of whether a blank matrix is available, and provides quantitative evaluation of MEs. When none of these approaches gives acceptable results in terms of low recovery and low precision and accuracy [195], the standard addition method or the use of a co-eluting internal standard are the only ways to obtain accurate quantitation.

## Figures and Tables

**Table 1:** Methods for the evaluation of matrix effects.

Name of the Method	Description of the Method	Limits	References
Post-column infusion method	The post-column infusion method provides a qualitative assessment of matrix effects. It permits the identification of the retention time zones in a chromatographic plot most likely to experience phenomena of ion enhancement or suppression. It consists in a constant flow through the LC-MS column of the mobile phase or blank, and the post column injection through a T-piece of the analyte standard. ME can be assessed as suppression or enhancement of the analyte signal in specific regions of the chromatogram.	Only qualitative results Inefficiency for highly diluted samples. The concentration of the analyte should be in the analytical range being investigated Laborious and time-consuming procedure, especially for multiresidue analysis Blank matrix not always available	[7,10,12,25,29,30,31,32,33,34,35,36,37,38,39]
Post-extraction spike method	In the post-extraction spike method, the response of the analyte in a standard solution is compared to that of the analyte spiked into a blank matrix sample at the same concentration. Deviations from the responses of the two solutions are identified as ion enhancement or suppression. This method is able to provide a quantitative assessment of matrix effect.	Blank matrix not always available	[26,40,41,42,43,44]
Slope Ratio Analysis	It allows a semi-quantitative screening of matrix effect. It exploits spiked samples and matrix-matched calibration standards at different calibration levels. This modified approach evaluates the same parameters obtained by post-extracion addition method in an entire selected range of concentrations instead of a single level.	Only semi-quantitative results	[28]
Relative MEs evaluation	It permits the evaluation of the variability of MEs lot by lot	Laborious	[3,26,45,46,47,48,49]

**Table 2:** Calibration approaches applied when the blank matrix is available.

Method	Theory or Mechanism	Advantages	Disadvantages	References
Matrix-matched calibration	External calibration presupposes the preparation of several samples from blank matrix spiked at different analyte concentrations before injection with linear calibrations calculated for each analyte.	It permits one to effectively overcome ME using a matrix the same as the sample	Laborious Time-consuming in relation to the necessity of processing of the blank matrix as the sample The test sample composition variance must be small Limitation in bioavailability of appropriate blanks	[46,57]
Isotope labeled internal standard	The use of internal standard implies the use of a substance with identical or similar ionization properties and very close retention time to that of the analyte. According to literature ^13^C-labelled IS mimes better than ^2^H-labelled IS the target analyte.	Better assay performance because they show identical behavior to the analyte in sample pretreatment Not time-consuming Particularly useful when homogeneous class of substances are analyzed	Expensive if SIL-ISs are used. For many compounds SIL-ISs are not commercially available Less suitable for multi residual analyses	[58,59,60,61,62,63,64,65]

**Table 3:** Calibration approaches applied when the blank matrix is not available.

Method	Theory or Mechanism	Advantages	Disadvantages	References
Standard addition	It requires that the analyte be spiked in same sample extract at different concentration levels.	Very effective	It requires large sample amount Very time-consuming	[23,67,68]
Background subtraction	The calibration curve is built by subtracting the background.	Useful for biological samples	Low reproducibility Less effective than other methods (lower sensitivity)	[23,66,69,70]
Surrogate matrix	It exploits surrogate matrixes such as neat solvent, stripped and artificial matrixes, that act as a blank-like matrix.	Effective and widely used approach It allows direct and sensitive quantification of analytes	Similar MS signal response of the analyte in both the surrogate and original matrix must be demonstrated	[23,55,70,71,72,73,74,75,76,77,78,79,80,81,82,83,84,85,86,87,88,89,90,91,92,93,94,95,96,97,98,99]
Surrogate analyte method	It requires stable-isotope-labeled standard as a surrogate analyte to allow calibration.	Very effective	Similar MS signal response of both the surrogate and original analyte must be demonstrated The utility of this method is limited by the availability of expensive and pure labeled standards.	[23,72,100]

**Table 4:** List of the protein removal (PPT) methodologies.

Matrix	Technique	Effectiveness	References
Infant food, plasma	Salting out	Residual ion suppression in ESI	[162,163]
Plasma, Urine	Precipitation with organic solvents	Not effective for other interference removal like phospholipids, lipids, aminoacids	[3,12,125,129,164,165,166]
Plasma, serum	ZnSO_4_ solution	Effective for PPT and decrease the phospholipids amount	[169,170]
Urine	Restricted access, volatile supramolecular solvents (RAM-VOL-SUPRAS)	Avoids or dramatically reduces the ME.	[171]
Fish muscle- Breast Milk	Protein-lipid removal filter cartridges (Captiva ND Lipids)	The best option to reduce ME in LC-MS applications	[172]

**Table 5:** List of the phospholipid removal methodologies.

Matrix	Technique	Effectiveness	References
Plasma	PPT	No extensive removal of PLs—ion suppression for many analytes	[125]
Plasma	Liquid-liquid extraction	Clean final extract but unacceptable analyte recovery especially for polar analytes	[125]
Plasma	Pure cationic exchange solid-phase extraction (SPE)	Cleaner extracts and reduced matrix effects compared to PPT	[125]
Plasma	Reversed-phase SPE	Cleaner extracts and reduced matrix effects compared to PPT	[125]
Plasma	Mixed-mode SPE	Best effectiveness because it combines the retention mechanisms of reverse-phase and ion exchange	[125]
Plasma	Solid supported liquid extraction (SLE)	Able to remove the majority of PLs through appropriate loading of buffers and eluting solvents	[165]
Plasma	Hybrid SPE-PPT(HybridSPE™, Ostro™, Phenomenex Phree™, Captiva™)	Effectively reduces the ME in biological samples	[130,178,179,180,181]
Urine	Restricted access, volatile supramolecular solvents (RAM-VOL-SUPRAS)	Avoids or dramatically reduces the ME.	[171]
Human plasma	Parallel Artificial Liquid Membrane Extraction (PALME)	Complete absence of ion suppression and high recovery percentage	[182]

**Table 6:** List of the lipid removal methodologies.

Matrix	Technique	Effectiveness	References
Porcine muscle-milk	LLE with hexane		[184,185]
High lipid foodstuff	Freezing lipid precipitation (LFP)	Effective sample clean-up	[186]
Milk	Lipid precipitation by zinc and tungsten salt solutions in acidic media	Good sensitivity	[187,188]
Milk	Dispersive solid phase extraction (d-SPE) by using a combination of PSA-C18 solid sorbent or PSA- Zirconium-dioxide	Very effective ME reduction	[189,191]
Fish muscle- Breast Milk	Protein-lipid removal filter cartridges (Captiva ND Lipids)	The best option to reduce ME in LC-MS applications. Effective also in protein removal	[172]
